# TREXIT Is Now: Should We Abandon the Transrectal Route for Prostate Biopsy? Yes

**DOI:** 10.1016/j.euros.2021.06.009

**Published:** 2021-07-19

**Authors:** Jeremy P. Grummet, Nicolas Mottet, Michael A. Gorin

**Affiliations:** a Department of Surgery, Alfred Health, Central Clinical School, Monash University, Melbourne, Australia; b Department of Urology, University Jean Monnet St. Etienne, Saint-Étienne, France; c Urology Associates and UPMC Western Maryland, Cumberland, MD, USA; d Department of Urology, University of Pittsburgh School of Medicine, Pittsburgh, PA, USA

Transperineal (TP) biopsy is safer than transrectal (TR) biopsy owing to its negligible risk of sepsis, but TR biopsy can be performed quickly under local anaesthetic (LA) in a doctor’s office. Until recently, TP biopsy had only been performed under general anaesthetic, taking longer to perform and clogging up operating theatres. This forced busy urologists to prioritise the convenience of TR biopsy over the clear benefits of TP biopsy.

We argue that TREXIT, the abandonment of TR biopsy in favour of TP biopsy, should be occurring globally now to prevent unnecessary harm to our patients while ensuring the highest degree of diagnostic accuracy. Sufficient evidence now exists that TP biopsy is safer in avoiding sepsis than the TR approach. Arguments prioritising practicality over patient safety have been dealt a fatal blow by the recently established use of TP biopsy under LA.

TR biopsy is increasingly causing life-threatening sepsis because of the ongoing rise of multidrug-resistant bacteria within rectal flora [1]. The rate of hospital admission reported for post-TR biopsy infection is as high as an astonishing 10% [2]. This is entirely iatrogenic and completely preventable. Fluoroquinolones have traditionally been the prophylactic drug of choice, but they are losing their effect as resistance rates rise [3]. Furthermore, use of this drug class for periprocedural prophylaxis has been suspended by the European Commission owing to the risk of long-term musculoskeletal and neurological complications [4] and there is a strong warning against fluoroquinolone use from the US Food and Drug Administration [5].

**Fig. 1:**
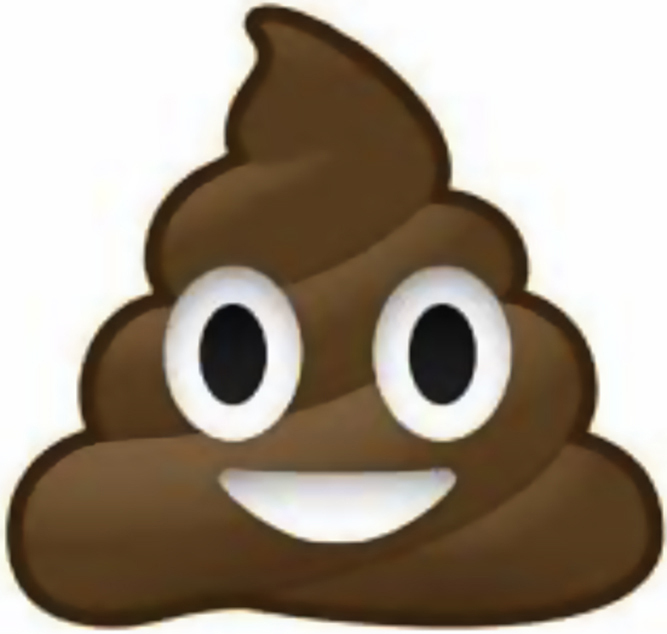
TR biopsy is dirty. Don’t be fooled by the friendly smile.

In response to increasing antibiotic resistance, clinicians have resorted to using targeted or multidrug prophylaxis for TR biopsy. While there is some evidence for the efficacy of these methods [6], they continue to ignore the underlying problem of using a “dirty” technique, as the biopsy trocar may be contaminated by faeces (Fig. 1). Thus, they go directly against the principles of antibiotic stewardship. While a benefit has been observed from attempting to cleanse the rectum with povidone-iodine for TR biopsy [7], this approach still relies on the use of targeted or multiple prophylactic antibiotics.

By contrast, TP biopsy, which is performed percutaneously, has a near-zero risk of sepsis. In addition, it does not require any such targeted or combinations of prophylactic antibiotics. Rather, it has been found that simple first-generation cephalosporin prophylaxis results in no sepsis. Furthermore, multiple TP biopsy series have now been published in which no antibiotics were given at all, still with zero rates of sepsis.

Seven randomised controlled trials including a total of 1330 patients have been published that include data comparing infection rates between TR and TP biopsy. These relatively small studies were not designed or powered to specifically address differences in infection rates. Despite this, when pooled, the trials showed a significantly higher rate of infection with TR biopsy (5.6% vs 3.3%; hazard ratio 1.81, 95% confidence interval 1.09–3.00) [7]. It should be noted that this included minor infections and not just sepsis. In addition, none of these trials compared TR biopsy using standard fluoroquinolone antibiotics to TP biopsy using a first-generation cephalosporin or no antibiotics at all, which single cohort series have shown to be safe (see below). Furthermore, a systematic review of 165 studies including more than 162 000 patients and comparing sepsis showed rates of 0.9% and 0.1% for TR and TP biopsy, respectively [8].

Multiple large single-cohort series have demonstrated the safety of TP biopsy when using either a simple first-generation cephalosporin as prophylaxis or no antibiotic at all. In 2017, a series of 577 consecutive patients underwent TP biopsy with a single dose of cephazolin. One patient developed prostatitis treated with oral antibiotics. There were no admissions for sepsis [9]. When the results of this study were updated to include 1194 patients, there were still no hospital admissions for infection [10]. A similar study of 485 patients using only cephazolin prophylaxis in 2018 reported four patients (0.8%) with infection, which included only one with sepsis [11]. In 2019, a larger study of 1287 patients undergoing TP biopsy (notably also under LA only) with single-dose cephalosporin had only a single patient with a positive urine culture and again there were no hospital admissions for infection. Notably, the rate of acute retention, often cited as a drawback of TP biopsy, was just 1.6% [12]. Similar results have been found for TP biopsy when no antibiotics are used at all. In a small study of 95 patients having TP biopsy under LA in 2019, only one patient received prophylactic antibiotics and there were no infections [13]. Similarly, in a 2020 study that included 177 patients undergoing TP biopsy under LA with no antibiotics, there were zero infections [14].

As mentioned, some of these and other large series have also shown the feasibility and diagnostic accuracy of performing TP biopsy freehand under LA only, for which an access cannula allows multiple trocar passes through only two skin puncture sites. In the study of 1287 patients by Stefanova et al [12] cited above, significant cancer was detected in 30% of cases and patients reported only mild discomfort. This year a multicentre study of 1014 LATP cases showed no cases of sepsis, a 39.4% detection rate for significant cancer, and a mean pain score of 3.1 [15] Another multicentre study of 1218 LATP cases published very recently showed a sepsis rate of 0.16% and a detection rate of 52% for significant cancer, with the majority of patients reporting little or no pain at all [16]. It should be noted that it has been shown that detection of significant prostate cancer with TP biopsy is equivalent to or better than TR biopsy, even when taking magnetic resonance imaging–targeted cores via either approach [17].

As the final nail in the TR coffin, accumulation of the evidence cited above has led to new recommendations in the European Association of Urology prostate cancer guidelines, which now favour the TP approach as the new standard of care. TREXIT is well under way, with an official National Health Service programme providing LATP biopsy training in the UK. TP biopsy is already common practice in Australia and is gaining momentum rapidly in North America.

  ***Conflicts of interest*:** Jeremy P. Grummet is co-founder of MRI PRO Pty Ltd. and has received honoraria from BK Ultrasound and travel grants from Biobot Surgical. Nicolas Mottet is a company consultant for Janssen, GE, BMS, Sanofi, Ipsen, AstraZeneca, Carrik, Arquer Diagnostics, Takeda, Bayer, and Astellas; has received speaker honoraria from Astellas, Pierre Fabre, Steba, Janssen, and Ferring; and has received fellowships and travel grants from Astellas, Ipsen, Sanofi, Janssen, and Roche. Michael A. Gorin is a paid consultant to Perineologic, KOELIS, and BK Medical ApS.
